# Preparation of Assembled Carbon Soot Films and Hydrophobic Properties

**DOI:** 10.3390/ma11112318

**Published:** 2018-11-19

**Authors:** Lei Zhao, Kang Zhao, Wei-Guo Yan, Zhifeng Liu

**Affiliations:** 1 School of Civil Engineering and Architecture, Wuhan University of Technology, Wuhan 430068, China; zhaolei197604@126.com; 2 School of Civil Engineering and Architecture, Xinxiang University, Xinxiang 453003, China; 3 School of Science, Tianjin Chengjian University, Tianjin 300384, China; luocl_2008@163.com; 4 School of Materials Science and Engineering, Tianjin Chengjian University, Tianjin 300384, China

**Keywords:** candle soot, self-assembly, hydrophobic, film

## Abstract

In this paper, a simple, inexpensive, and rapid method for the fabrication of controlled layer candle soot film has been reported by interface self-assembly and transferred method. The mechanism of candle soot self-assembly is explained and their morphology, elemental composition, optical, and wetting properties are characterized. The uniformity and thickness of prepared films especially depend on the concentration of candle soot mixed solution (alcohol and deionized water). The results show that the optimal concentration of candle soot solution is approximately ~0.2% wt/mL. In addition, the absorption spectra of the controlled-layer candle soot films are determined by the number of layers and the surface morphology. The hydrophobic properties of candle soot films are closely related to their layer number. When these films reach to the fourth layer, the water contact angle and roll-off angle are measured as 142° ± 2° and 6°, respectively. The controlled assembly CS films have the potential application in photo/electrocatalysis, solar cells, lithium-ion batteries, and water splitting.

## 1. Introduction

Over the decades, carbon nanomaterials (e.g., carbon nanotube and graphite) have shown wide applications, such as high-speed transistors, transparent electrodes, super-capacitors, and super-sensitive bio-sensors due to their novel physical, chemical, and mechanical properties [1,2,3,4]. As a member of carbonous family, carbon soot (CS), as an environmentally-friendly and abundant nano-material, has demonstrated the advantages in production scalability over graphite and carbon-nanotube in their synthesis. However, CS has received little attention on exploring its potential applications due to the opacity, inhomogeneity, and poor conductivity. Up to 2012, Xu Deng employed CS as a template for fabricating transparent and robust superhydrophobic coating [5]. Interestingly, due to the particularity of structure, these surfaces of the CS template exhibited superamphiphobic properties include superhydrophobic properties and oleophobic performance. Superamphiphobic functionalities of CS make them useful in many applications including oil/water separation, anti-icing, energy-saving buildings, and corrosion resistance [6,7,8,9,10,11,12]. 

Although CS templated silica surfaces have exhibited outstanding performance in many applications, the substrate must withstand high temperatures because CS templates are collected by means of flames. This has severely limited CS templates for the wide application in solar cells, antireflection surface, and oil/water separation. Recently, research showed that CS nanoparticles can dissolve in different solvents such as ethanol, isopropanol, and acetone. CS solution was sprayed on a glass to fabricate superhydrophobic surfaces [13]. However, the deposition thickness of the CS films is very difficult to accurately control by adjusting spraying time and the concentration. To address this problem, a simple, inexpensive and rapid method is proposed to fabricate large-scale and uniform CS films by interface self-assembly and transferred method. 

In this paper, an interface self-assembly and transferred method used to prepare large-scale and uniform CS films. During self-assembly, the concentration of CS solution is an important and key factor in fabricating CS films. The results show that the optimal concentration of CS solution is approximately ~0.2% wt/mL. CS self-assembly films with different layers are prepared by transferred method. In addition, the absorption intensities of CS films gradually improve with the increase of layers. The wettability of CS films is closely related to the number of CS layer. When CS films layer reaches to the fourth layer, the hydrophobic properties present optimum result. The water contact angle and roll-off angle are 142 ± 2° and 6°, respectively. The self-assembly mechanism of CS nanoparticles will be explored in detail. The controlled assemble CS template has the potential application in photo/electrocatalysis, solar cells, lithium-ion batteries, and water splitting.

## 2. Materials and Methods

### 2.1. Materials

**Figure 1:**
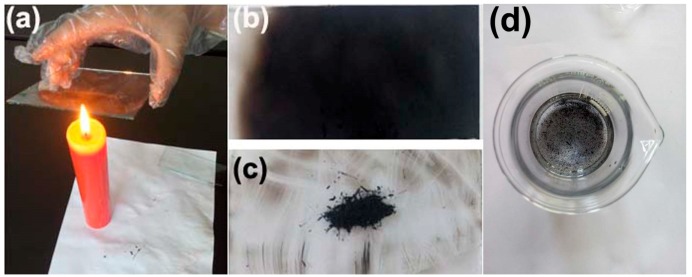
Preparation of CS solution: (**a**) a glass slide is held in the flame of a candle for 2 min; (**b**) a CS layer is deposited; (**c**) gather approximately 200 mg CS from glass slide; and (**d**) CS mixed solution.

Glass slides (7.5 × 2.5 cm^2^) and indium tin oxides (ITO) (1.5 × 2.5 cm^2^) are cleaned in ultrasound bath in deionized water, acetone, and isopropyl alcohol for 10 min respectively and dried under clean nitrogen flow. A glass slide is held on the flame of a candle for 2 min until a CS layer is deposited, as showed in Figure 1a,b. Then, gathering approximately 200 mg the CS from glass slide in Figure 1c. Highly stable homogeneous dispersions of the CS are prepared by simply mixing 20 mg CS in 200 mL alcohol and water solution (the ratio is 1:1), as showed in Figure 1d. The CS solution is further diluted to 1, 0.5, 0.4, 0.3, 0.2, and 0.1% wt/mL respectively. 

### 2.2. Fabrication of CS Self-Assembly Films

**Figure 2:**
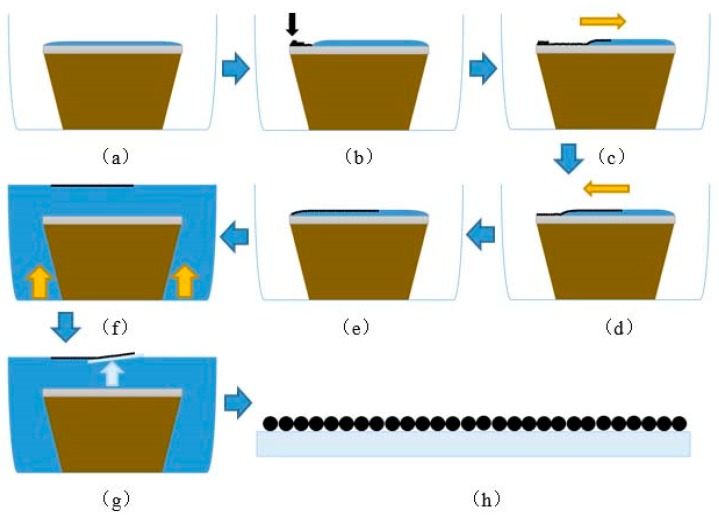
The fabrication process of interface self-assembly and transferred method. (**a**) water film on the substrate; (**b**) the mixed CS solution is first injected into water film; (**c**–**e**) a CS film formed at the air/water interface; (**f**) detach CS onto the water; (**g**) catching up the CS film by the substrate; (**h**) the CS film on the substrate.

A given volume of deionized water is dropped on a hydrophilic glass slide. The deionized water spreads onto the whole glass slide and forms a water film in Figure 2a. The 75 μL CS mixed solution slowly injects onto the edge of glass slide by a micropipette, as is showed in Figure 2b. The CS spreads onto the water surface in Figure 2c–e. The CS self-assembly films detach from the glass slide and float on the water surface in several minutes after injecting water into the container, as is shown in Figure 2f. The floating CS films can be picked up by ITO in Figure 2g. At last, samples are placed in thermostat and dried for 10 min, until the CS films consolidate onto the surface of ITO, as is showed in Figure 2h.The CS films with different layers can be transferred on the substrate by repeating the previous steps, the experimental process shows in Figure 2. 

### 2.3. Characterization

The surface morphology of the CS films is observed by using a JEOL JSM-7800F scanning electron microscope (SEM, JEOL Ltd., Tokyo, Japan) operated at an accelerating voltage of 5 kV. The chemical characterization of the CS film is carried out by using energy dispersive spectroscopy (EDS, JEOL Ltd., Tokyo, Japan) and Flourier transformation infrared spectroscopy (FTIR, Thermo Scientific, Waltham, MA, USA). DU-8B UV–vis double-beam spectrophotometer (Shang HaiSunny Hengping Scientific Instrument Co., Shanghai, China) is used for optical absorption capabilities examination. The static water wettability of a surface of CS film with different layers is measured by contact angle tester (DSA series, Mouser Electronics, Shanghai, China). 

## 3. Results and Discussion

### 3.1. Interface Self-Assembly of CS Solution with Different Concentration

**Figure 3:**
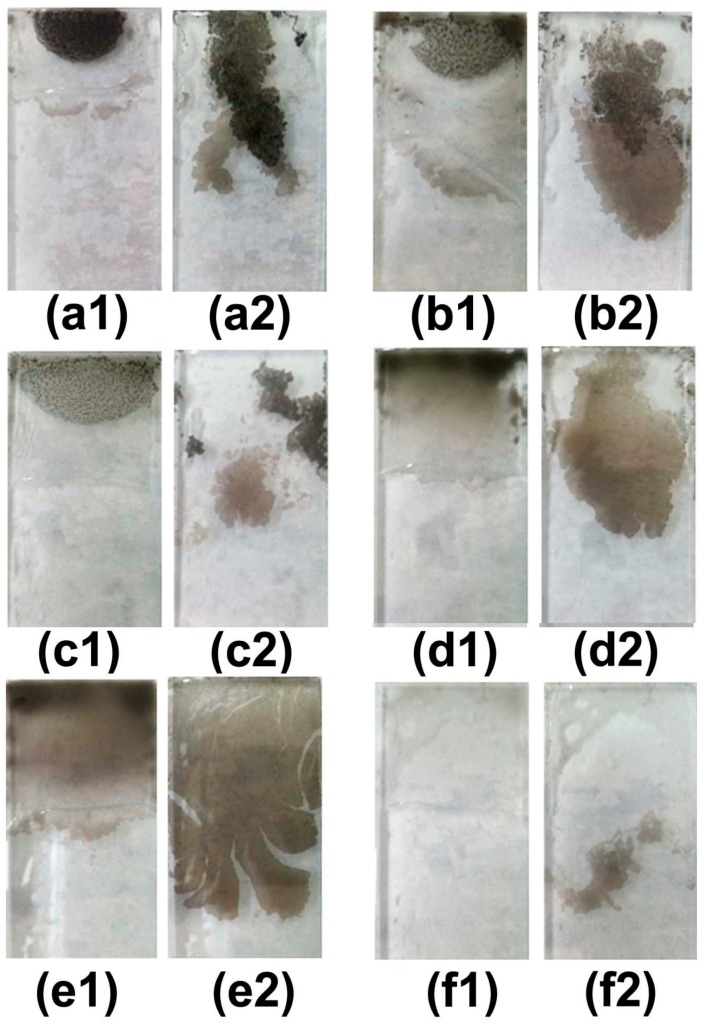
The different concentration of CS solution used to prepare CS films by interface self-assembly and transferred method: (**a1**,**a2**) 1%; (**b1**,**b2**) 0.5%; (**c1**,**c2**) 0.4%; (**d1**,**d2**) 0.3%, (**e1**,**e2**) 0.2%; and (**f1**,**f2**) 0.1% wt/mL, respectively.

Interface self-assembly is an effective method to fabricate some novel structures [14,15,16]. In our experiments, the different concentration of CS solution with 1, 0.5, 0.4, 0.3, 0.2, and 0.1% wt/mL respectively used to self-assemble. When the concentration of CS solution is 1%, CS particles are very difficult to fully mix with pure water and alcohol (the ratio is 1:1). So, during the self-assembly process, the CS particles gather together, and then result in the nonuniformity of CS films in Figure 3(a1). Although the CS particles can float on the surface of water under the effect of alcohol evaporation, self-assemble CS films are very inhomogeneous, as is showed in Figure 3(a2). When the concentration of CS solution is diluted to 0.5, 0.4, and 0.3%, the quality of CS self-assembly films has weak improvement than the CS solution of 1% in the Figure 3(b1–d2). When the concentration of CS solution is further diluted to 0.2%, it is found that CS particles are easily dissolved in mixed solution of pure water and alcohol. CS particles have no obvious aggregation during self-assembly. The large-scale CS self-assembly films float on the surface of water, as is showed in Figure 3(e1,e2). When the concentration of CS solution further diluted to 0.1%, the area of CS self-assembly films became smaller than the concentration of 0.2% in Figure 3(f1,f2). Above all, the ideal concentration of CS solution is approximately ~0.2% wt/mL, CS solution can assemble large-scale CS films.

### 3.2. Time Evolution of CS Self-Assembly Film Formation

**Figure 4:**
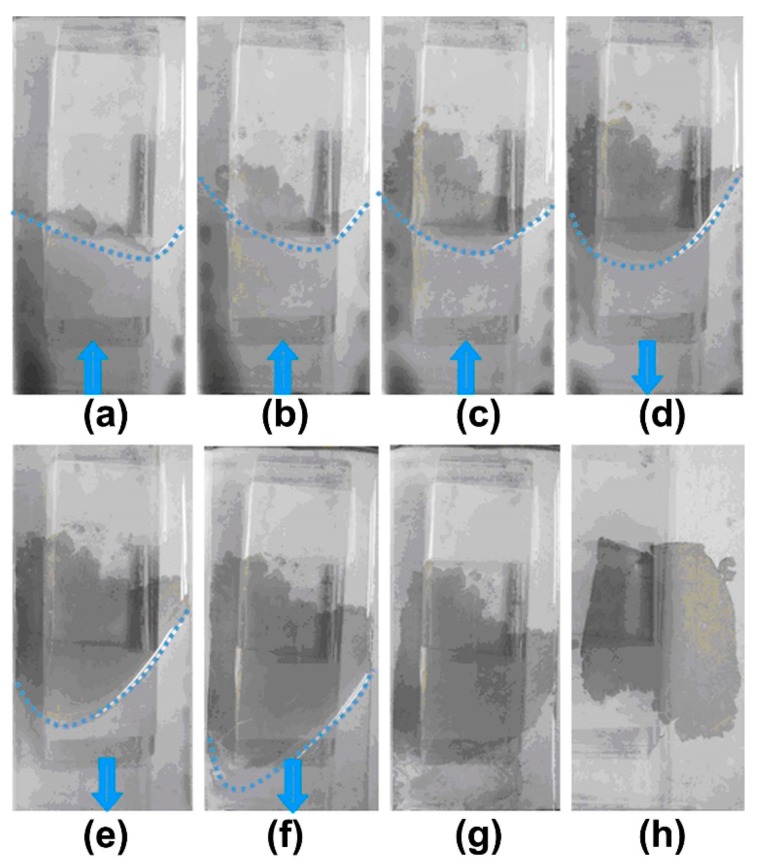
The self-assembly process of CS solution with the concentration of 0.2% wt/mL: (**a**) 10 s, (**b**) 30 s, (**c**) 60 s, (**d**) 120 s, (**e**) 180 s, (**f**) 240 s, (**g**) 360 s, (**h**) 600 s.

In our experiment, the CS solution with concentration of 0.2% wt/mL is used to fabricate large-scale CS films. The self-assembly process of CS on the interface is shown in Figure 4a–h. The about 30 mL deionized water drops on the hydrophilic glass slide and spreads the whole surface of the glass slide due to hydrophilic effects. The 50 uL CS solution drops on one side of the glass slide, the water is pushed to the other end of the glass slide due to the volatilization of alcohol, as is showed in Figure 4a. At the same time, the CS solution spreads onto the surface of water film, and then self-assembles into the membrane under the influence of the surface tension (blue dashed line in Figure 4a is the position of the CS mixed solution and water film junction, the blue arrows indicate the movement of the water film direction). As time went by, more and more CS solution gradually floats on the surface of the water, as showed in Figure 4b,c. With alcohol evaporation on the glass slide, the water film spreads slowly back and CS particles float on the surface of water film, as is shown in Figure 4d–f. When the water film returns back to its original position, the CS films fully formed, as showed in Figure 4g. After injecting a certain amount of water into the container, self-assembly CS film floats on the surface of water in container. 

### 3.3. Optical Properties of CS Self-Assembly Films with Different Layers

**Figure 5:**
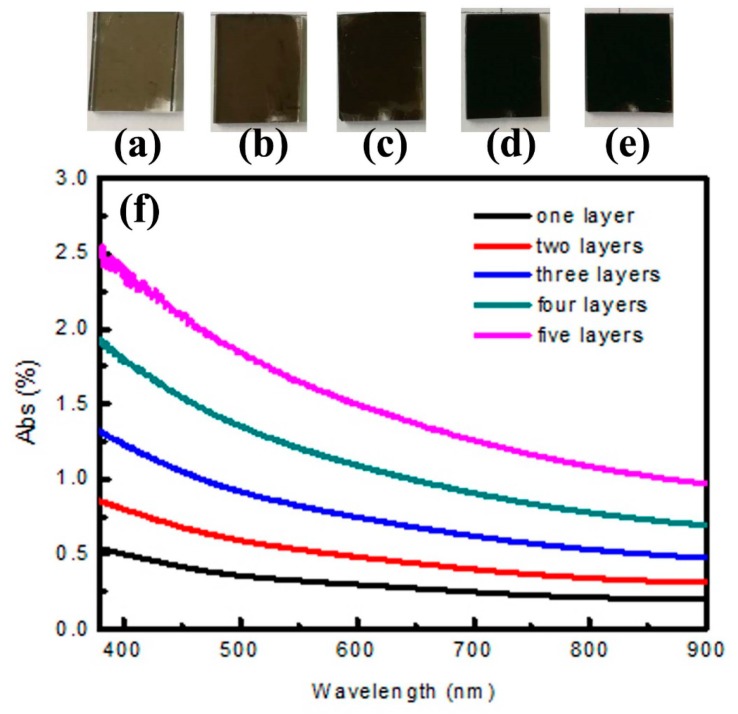
Optical properties of CS self-assembly films with different layers: (**a**–**e**) the photographs of five samples respectively; (**f**) absorption spectra of five samples.

The samples are large-scale CS self-assembly films with the different layers, as is showed in Figure 5a–e. It is obviously seen that the color of the samples became deeper and deeper with the increase of the layer. For the first CS self-assembly film, this sample presents high light transmission and good homogeneity in Figure 5a. When the second CS self-assembly film coated on the sample, it is seen that the homogeneity of sample remains fine from Figure 5b. However, light transmittance is significantly lower than the first one. When CS self-assembly films add to the fifth layer, the sample is completely black in Figure 5d.

To accurately compare the optical properties of CS films with different layers, the absorption spectra of the samples are examined by DU-8B UV–vis spectrophotometer. For the first CS self-assembly films, we can see that the absorption intensity does not exceed 0.5% in the visible region from the Figure 5f (the black line). With the increase of the layers, the absorption intensity increases linearly. When the number of CS film layer adds to the fifth layer (the pink curve corresponds to the five-layer film), the max absorption intensity at 380 nm reaches to 2.5%. Therefore, this environmentally-friendly CS films can bring the potential application in photo/electrocatalysis, solar cells, lithium-ion batteries by adjusting the number of layers.

### 3.4. Composition and Morphology of CS Self-Assembly Films with Different Layers

**Figure 6:**
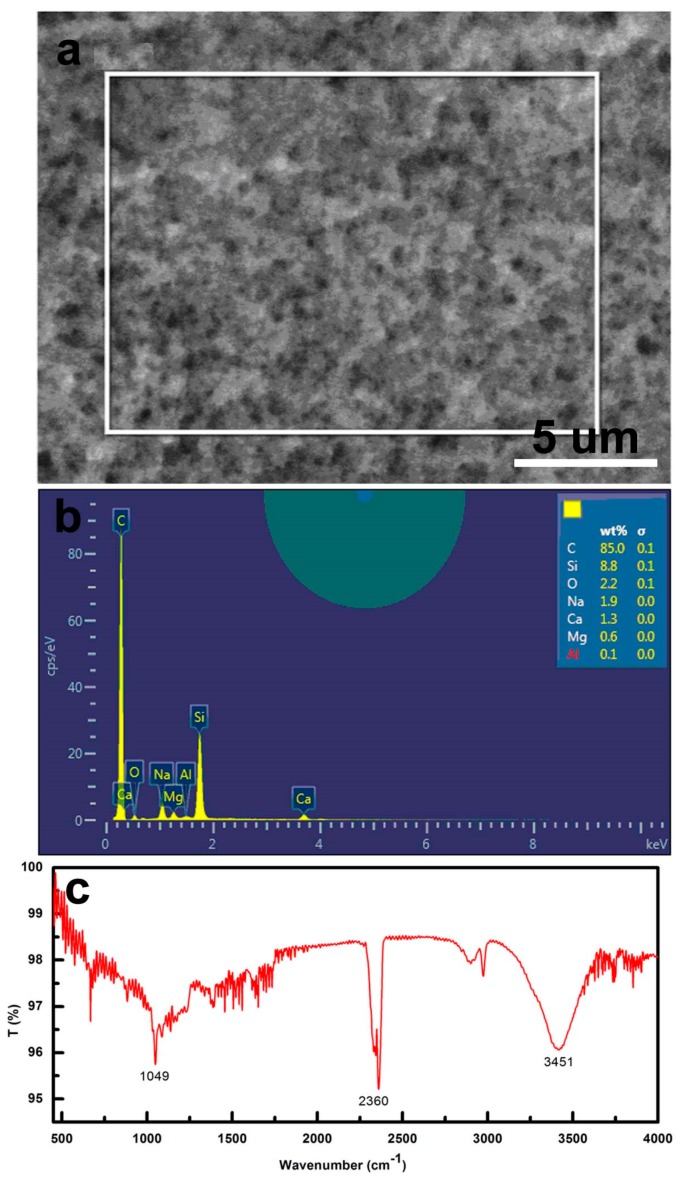
Chemical characterization of the CS film: (**a**) test area on CS film of EDS; (**b**) the chemical characterization (EDS); (**c**) FTIR of CS film.

The typical SEM image (top view) of CS self-assembly film is showed in Figure 6a. From the image of Figure 6a, we can see that the CS self-assembly film shows good homogeneity. The chemical characterization of the CS film is carried out by using energy dispersive X-ray spectroscopy (EDS) and Flourier transformation infrared spectroscopy (FTIR). The results show that the sample is composed of carbon (weight% = 85.0), oxygen (weight% = 2.2) and silicone (weight% = 8.8) as the main constituents with carbon having the highest percentage, as is showed in Figure 6b. From FTIR, there are three main peaks at 1049, 2360, and 3451 cm^−1^ respectively in Figure 6c. The peak at 3451 cm^–1^ is associated with –OH vibration of water. (C–H) stretching bands occur between 3300 cm^−1^ and 2800 cm^−1^. The peaks at 2883 cm^−1^ corresponds to aliphatic (C–H) stretching band. The peaks between 1040 and 1240 cm^−1^ indicate (C–O) bonds of ethers, carboxylic acids, and polysaccharide.

**Figure 7:**
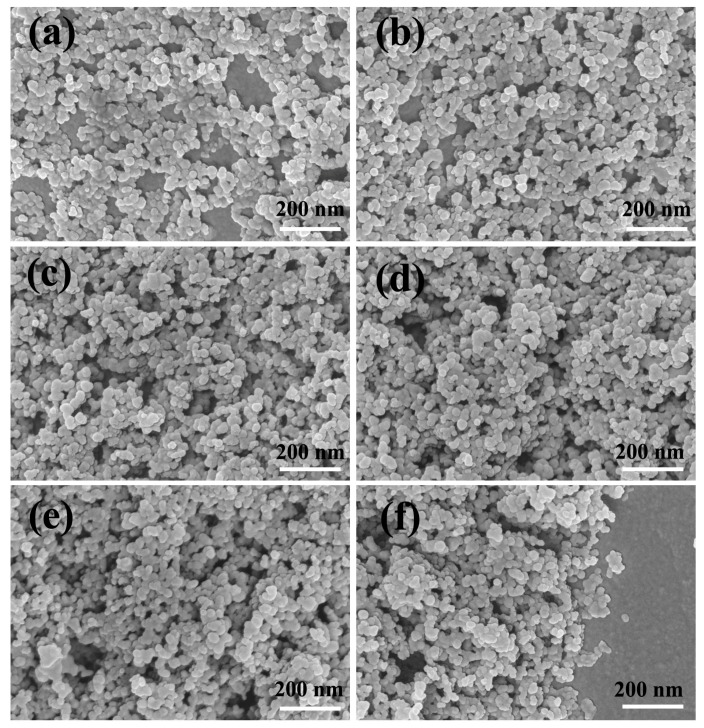
SEM images of CS films: (**a**) the first-layer CS film; (**b**) the second-layer CS film; (**c**) the third-layer CS film; (**d**) the fourth-layer CS film; (**e**) the fifth-layer CS film; (**f**) the lateral SEM image of the fifth-layer CS film.

Figure 7 shows the SEM images of CS self-assembly films with different layers. CS films consist of carbon particles with a typical diameter of 10 to 100 nm (Supplemental Information, Figure S2) [17]. From the images of Figure 7a–f, it is can be seen clearly that the CS nanoparticles become closer and closer with the increase of the number of layers. There are obvious defects among the CS films in Figure 7a. On the one hand, CS nanoparticles mostly cover the surface of the substrate, on the other hand, the CS nanoparticles appear “heaped and piled” phenomenon. Although CS nanoparticles solution is oscillated in ultrasonic oscillator for 2 h, the “heaped and piled” effect of CS nanoparticles gets no better than 30 min. This implies that CS nanoparticles self-aggregate in the mixed solution of alcohol and water. After the second-layer CS film is transferred to the surface of the first-layer, there is a marked increase of the coverage of CS nanoparticles than the first-layer, as is showed in Figure 7b. When CS films add to the third-layer, more CS nanoparticles covered on the substrate in Figure 7c. Surface roughness of third-layer CS film obviously improves compared with those of the first-layer and second-one. In order to further enhance the surface roughness of CS film, the fourth and fifth-layer CS films are transferred again by repeating the previous steps. Figure 7f,e shows that surface morphology of the fourth CS film is very similar with the fifth-layer one. However, we found that the stability of the fifth-layer CS film is relatively unstable, which is easily fallen off. Figure 7f illustrates a side view of the fifth-layer CS film.

**Figure 8:**
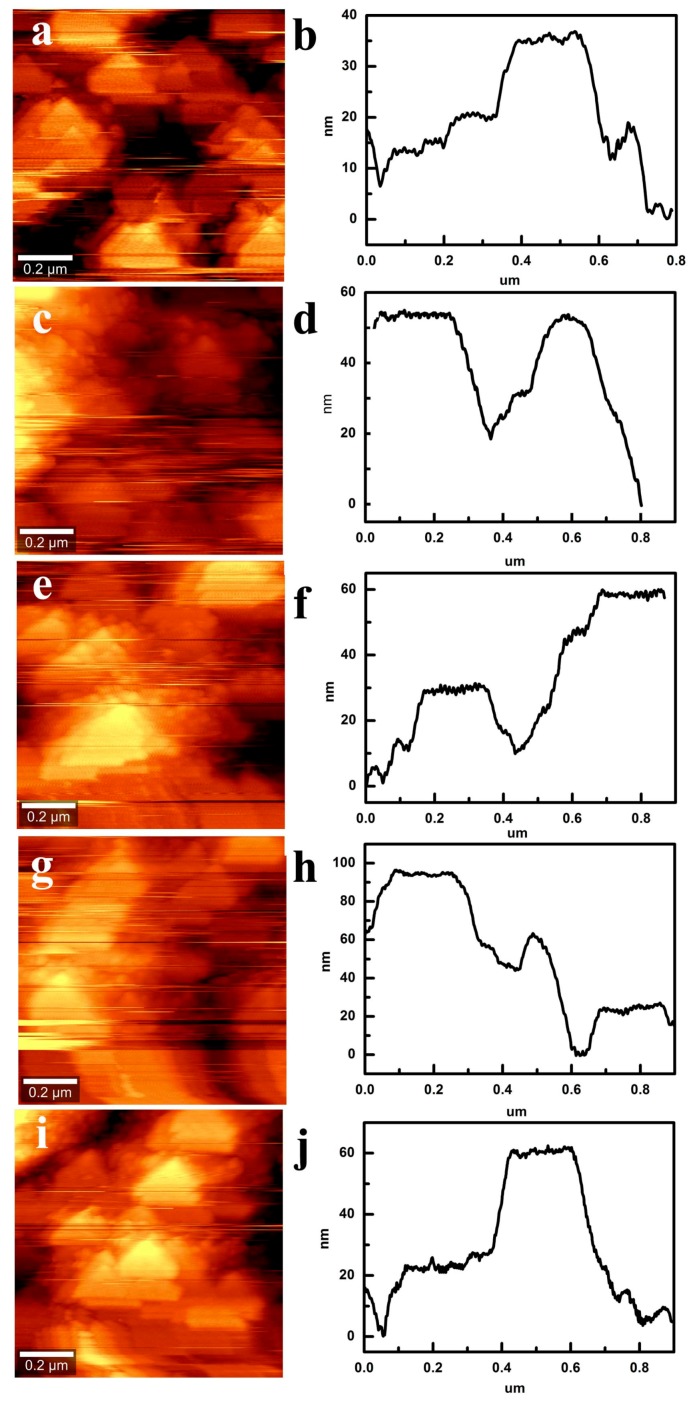
AFM of CS films with different layers: (**a**,**b**) the first-layer CS film; (**c**,**d**) the second-layer CS film; (**e**,**f**) the third-layer CS film; (**g**,**h**) the fourth-layer CS film; (**i**,**j**) the fifth-layer CS film.

To evaluate the roughness of CS films with different layers, AFM is used to characterize the surface microstructure, as is showed in Figure 8a–j. From AFM images in Figure 8a,b, it is observed that the height difference between CS particles is about 35 nm. The height difference between CS particles refers to surface roughness. With the increase of the number of layers, surface roughness of CS films gradually enhances. When the number of CS layers reaches the fourth layer, height difference between CS particles approaches 100 nm, as is showed in Figure 8g,h. However, after the fifth-layer CS film coats on the fourth-layer one, surface roughness reduces by about 60 nm in Figure 8i,j. Surface roughness is a key factor to evaluate wettability of hydrophobic material.

### 3.5. Hydrophobic Performance of CS Self-Assembly Films with Different Layers

**Figure 9:**
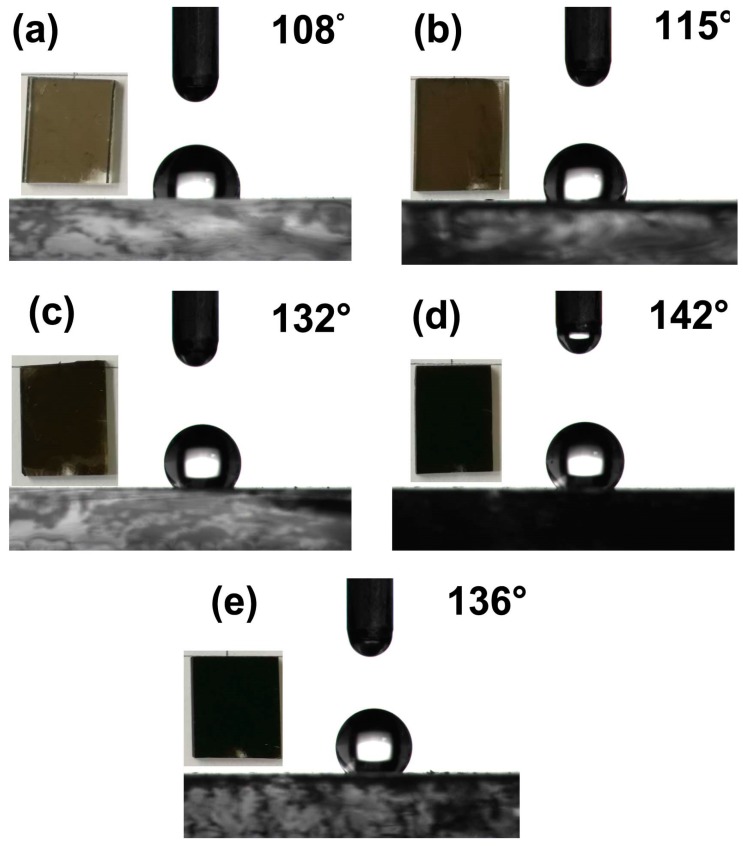
Contact angles: (**a**) the first-layer CS film; (**b**) the second-layer CS film; (**c**) the third-layer CS film; (**d**) the fourth-layer CS film; (**e**) the fifth-layer CS film.

As it is well known, the surface roughness of samples play a very significant part in the wetting properties. The contact angle (CA) and roll-off angle are the criterion to judge the wettability of the surface of as-prepared samples. To obtain hydrophobic surface, we enhance the surface roughness by adding the number of CS films layer. Figure 9 illustrates the water contact angles of CS films with different layers. The contact angle of the first-layer CS films is about 108°, as is showed in Figure 9a. As can be seen from the image of Figure 8a, surface roughness of the first-layer CS films is small, which leads to the small contact angle. Figure 9b shows the CA optical images of the second-layer CS films, which is 115°. The CA of the second-layer CS films has an obvious improvement than the first one. In the Wenzel regime, surface roughness can promote wettability of material. Therefore, we prepare the third-layer, fourth-layer, and fifth-layer CS films. The results show their CAs are 132°, 142°, and 136° respectively, as is showed in Figure 9c–e. Compared with the previous CS films, the hydrophobic properties have an obvious improvement, especially the fourth one. However, the hydrophobicity of material not only depends on the surface chemistry but also the roughness of surface. When the fifth-layer CS film coated on the sample, the low surface energy leads to poor adhesion of CS particles. Therefore, the CA of the fifth CS films reduced to 136°, as is shown in Figure 9e. To further test the stability of CS films, all samples are placed in ambient environment for seven days. The CAs of CS films with different layers are measured again. The results show that CAs of CS films with different layers are slightly lower than the original samples. The CAs of CS films from one layer to five layers are 105°, 110°, 128°, 138°, and 110° respectively (Supplemental Information, Figure S1).

## 4. Conclusions

In summary, a simple and inexpensive method is proposed for achieving large-scale self-assembly CS films with different layers on ITO substrate. The result shows the concentration of CS solution plays an important role during CS self-assembly, which is approximately ~0.2%. The layers of CS films can be controlled by the transferring times. Light transmittance of CS films increases to 2.5% with increasing of the number of CS films layer. In addition, the contact angle of the fourth CS film is the maximum compared with others and the contact angle and roll-off angle are measured as 142° ± 2°and 6° respectively. The CS films with controlled layers can be fabricated on different substrates, such as ITO, Si, and GaN. This method makes them useful in many applications including oil/water separation, anti-icing, energy-saving buildings, and corrosion resistance.

## Supplementary Materials

The following are available online at http://www.mdpi.com/1996-1944/11/11/2318/s1, Figure S1: The CAs of CS films with different layers after placing in ambient environment for seven days: (a) the first-layer CS film; (b) the second-layer CS film; (c) the third-layer CS film; (d) the fourth-layer CS film; (e) the fifth-layer CS film, Figure S2: The high resolution picture of SEM of candle soot.
