# Supplementary material for: Preparation of Assembled Carbon Soot Films and Hydrophobic Properties
Source: Materials (Basel). 2018 Nov 19;11(11):2318. doi: 10.3390/ma11112318 (PMC6266347; doi:10.3390/ma11112318)
Supplement: Supplementary file 1 [file materials-11-02318-s001.pdf]

Supplemental Information:

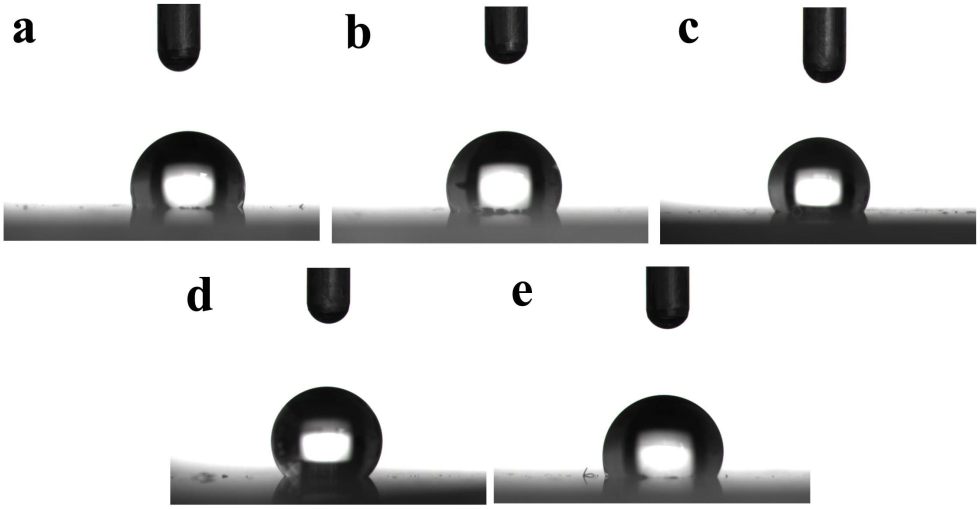

Figure S1. The CAs of CS films with different layers after placing in ambient environment for seven days: (**a**) the first-layer CS film; (**b**) the second-layer CS film; (**c**) the third-layer CS film; (**d**) the fourth-layer CS film; (**e**) the fifth-layer CS film.

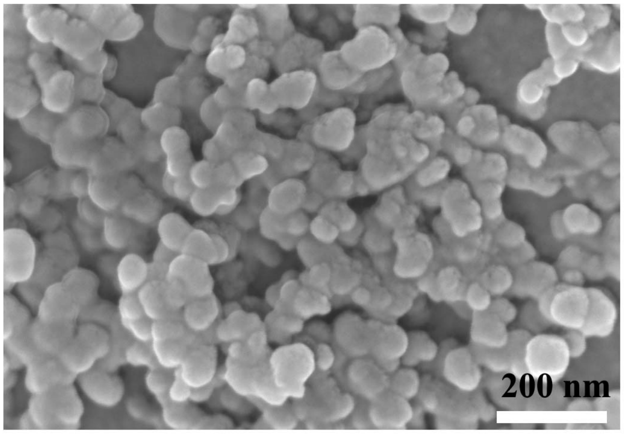

Figure S2. The high resolution picture of SEM of candle soot.
